# Using Artificial Neuro-Molecular System in Robotic Arm Motion Control—Taking Simulation of Rehabilitation as an Example

**DOI:** 10.3390/s22072584

**Published:** 2022-03-28

**Authors:** Jong-Chen Chen

**Affiliations:** Information Management, National Yunlin University of Science and Technology, Yunlin 64002, Taiwan; jcchen@yuntech.edu.tw; Tel.: +886-921-717-966

**Keywords:** robot, computational intelligence, evolutionary learning, self-organizing learning, robotic arm, simulation

## Abstract

Under the delicate control of the brain, people can perform graceful movements through the coordination of muscles, bones, ligaments, and joints. If artificial intelligence can be used to establish a control system that simulates the movements of human arms, it is believed that the application scope of robotic arms in assisting people’s daily life can be greatly increased. The purpose of this study is to build a general system that can use intelligent techniques to assist in the construction of a personalized rehabilitation system. More importantly, this research hopes to establish an intelligent system that can be adjusted according to the needs of the problem domain, that is, the system can move toward the direction of problem-solving through autonomous learning. The artificial neural molecular system (ANM system), developed early in our laboratory, which captured the close structure/function relationship of biological systems, was used. The system was operated on the V-REP (Virtual Robot Experimentation Platform). The results show that the ANM system can use self-learning methods to adjust the start-up time, rotation angle, and the sequence of the motor operation of different motors in order to complete the designated task assignment.

## 1. Introduction

Humans are the most exquisite works of art created by God. This is because the human body has the most complex and delicate structure. Under the control of the brain, this structure completes its movements through the coordination of muscles, bones, ligaments, and joints. If intelligent control can be applied to bionic robotic arms, it is believed that it should be able to expand the application scope of humanoid robotic arms. A number of scholars [1,2,3,4,5,6,7] showed that robotic therapies may be useful for limb rehabilitation and have an additional benefit over the control therapy. 

Ritchie et al. [8,9] developed a passive robotic arm capable of recording and facilitating the movement of the upper extremity to aid in the rehabilitation of the upper extremity after stroke. Another line of scholars [10,11] have suggested that robotic-assisted therapy may help improve arm and function in patients with cervical spinal cord injury. In addition to using robot-assisted therapy, another group of scholars [12,13,14,15,16] advocates the use of exoskeletons for rehabilitation. They believe that the exoskeleton approach is easy to use, highly acceptable, and relatively less uncomfortable for patients.

The above-mentioned systems are basically designed for a specific group of needs. For example, some rehabilitation systems are specially designed for patients who have suffered a stroke while others are for patients with cervical spinal cord injury. If further classified, some systems are designed for the rehabilitation of the upper limbs, and some systems are designed for the rehabilitation of the lower limbs. Some are even designed for functional rehabilitation of the palm and fingers. There is no doubt that all of the above-mentioned systems can perform very well and show excellent performance in the rehabilitation of a certain function for a specific ethnic group.

The purpose of this study is not to build a state-of-the-art rehabilitation system but to use it as an experimental testbed to build a general system that can use intelligent techniques to assist in the construction of a personalized rehabilitation system. More importantly, this research hopes to establish an intelligent system that can be adjusted according to the needs of the problem domain, that is, the system can move toward the direction of problem-solving through autonomous learning. We all know that as far as a normal healthy person is concerned, he (she) can perform most actions that ordinary people can perform. However, for a patient, what appears to be a relatively simple action to a healthy normal person may be quite difficult for him or her. During the rehabilitation process, progressive rehabilitation becomes very important for a patient. Most importantly, the patient must be careful to avoid the possibility of secondary injury. Roughly speaking, progressive rehabilitation emphasizes that patients must be under the timely guidance of professional physicians at different stages in order to achieve a truly effective rehabilitation effect without harm. In other words, professional physicians must carefully evaluate the patient’s rehabilitation and recommend appropriate and feasible actions. At different stages of rehabilitation, physicians guide patients in a timely manner in a step-by-step manner to achieve a progressive recovery effect without injury. In the case of using robots to assist patients in rehabilitation, how can doctors clearly express and recommend rehabilitation actions in the control of robots? From the control of the robot, how to generate the rehabilitation action suggested by the doctor to the patient is a relatively more difficult problem than using the robot to assist the patient in rehabilitation.

The traditional way to solve the above problem is to use the inverse kinematics method. This method is to move the manipulator (usually the endpoint) to a specified position through inverse kinematics calculation, thereby controlling the motor angle, activation time, and sequence of each joint. There are two possible problems with this approach. The first is that suitable or feasible answers could be found only through precise calculations and deductions. The second problem is that when a patient is assigned a specific arm movement trajectory due to individual needs, it may be quite difficult to find the answer through inverse kinematics calculations. 

The working mechanism of the ANM system proposed in this research is not to find these answers by means of inverse kinematics calculations but to obtain them through autonomous learning. The advantage of this approach is that when needs change (e.g., when the patient or trajectory changes), it does not need to be derived again through precise calculations or mathematical formulas. The trajectory experiments used in this study are designed to address this question. 

In response to the above requirements, whether or not a system has continuous learning ability plays an important role. The ANM system used in this study [17,18] differs from traditional neural networks in that it emphasizes information processing within neurons to capture the gradually changing features of biological structure/function. Through evolutionary learning and autonomous learning, the system is shaped into the special input/output information processors required by the problem domain. The system has been shown to be effective in different fields, such as robotic maze navigation [17,18], chopstick robotic applications [19], and snake-like robotic locomotion [20]. 

Different from previous studies, the target point of this study is not a single point but a series of points with a time sequence (in this study, it is the movement trajectory of a robotic arm). During the learning process, it must find the maximum possible similarity between two moving trajectories with different lengths. Therefore, when compared with previous studies, the learning difficulty of this study is also relatively high. 

## 2. Materials and Methods

### 2.1. The ANM System

**Figure 1:**
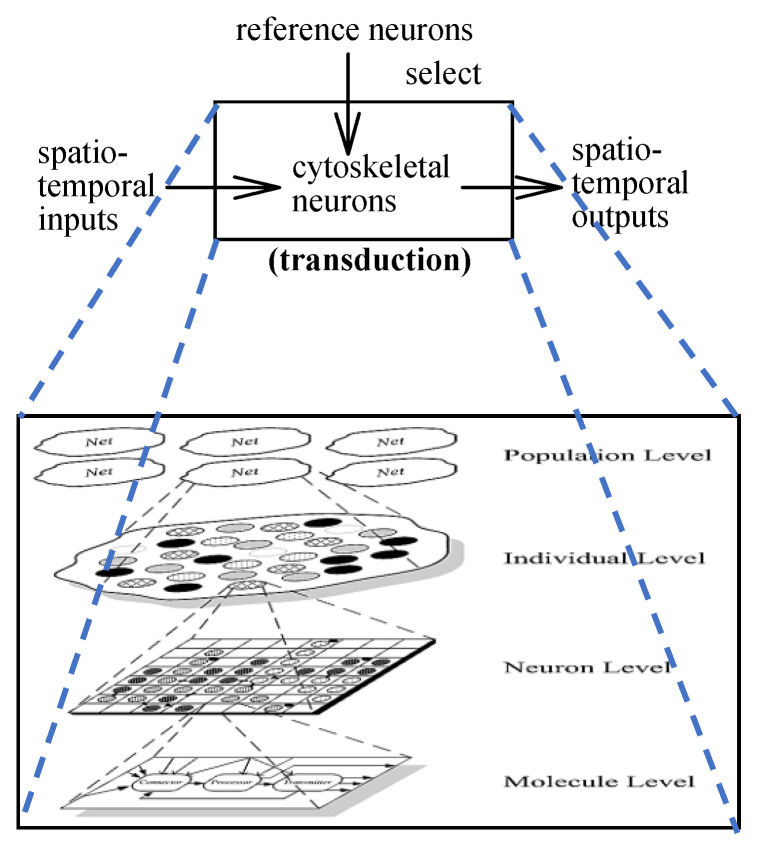
Architecture of the ANM system.

The ANM system mainly consists of a group of control neurons (or Reference Neurons) and a group of information processing neurons (or Cytoskeletal Neurons), which constitute the core processing elements of the system (Figure 1). Control neurons are divided into high-level control neurons (High-Level Reference Neurons) and low-level control neurons (Low-Level Reference Neurons) in this architecture. There are a total of 256 information processing neurons, which are divided into eight sub-networks. Each low-level control neuron simultaneously controls one of the information processing neurons in each sub-network (note: these neurons have a neuron-like dynamic structure). Therefore, under this design architecture, activating a low-level control neuron will simultaneously activate all the information processing neurons in the eight sub-networks that it controls with similar internal neuron dynamics.

The neuronal connections between the low-level control neuron and the information processing neuron are fixed and do not change over time. High-level control neurons are mainly responsible for activating different combinations of low-level control neurons. The neuronal connections between the high- and the low-level control neuron might change as learning progresses (noted that only selected information processing elements can participate in receiving input information from sensory neurons. Correspondingly, only selected information processing elements can respond to neuron outputs).

### 2.2. Conceptual Architecture of Information Processing (IP) Neuron

**Figure 2:**
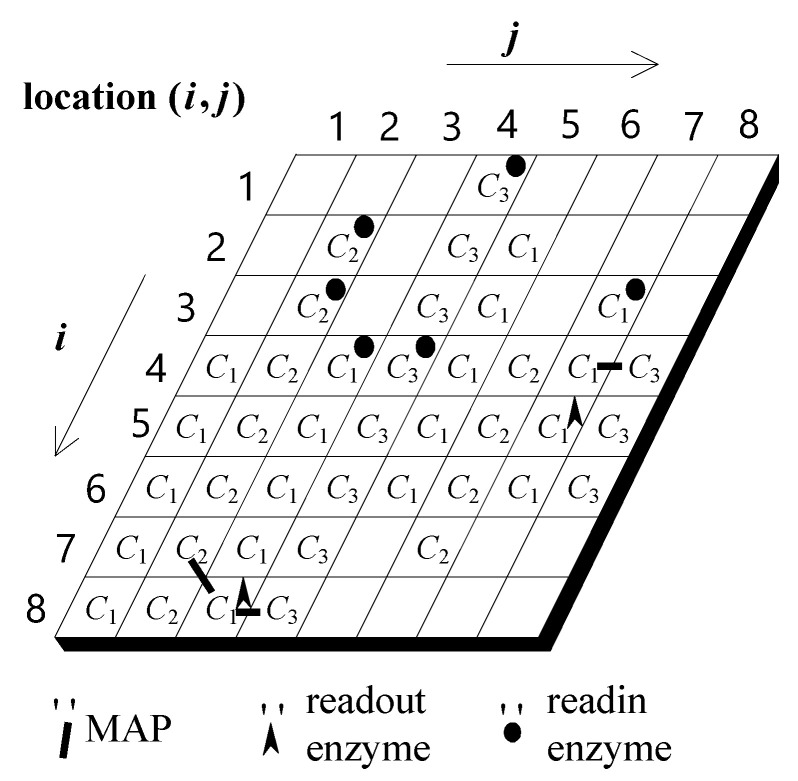
Conceptual architecture of an IP neuron.

As shown in Figure 2, we model the information processing of an information processing unit using a two-dimensional cellular automaton. Each grid represents a basic molecular unit. It is currently assumed that there are three different types of basic molecules (denoted *C*_1_, *C*_2_, and *C*_3_, respectively). The role of each basic molecule is to transmit and integrate signals to and from its eight neighboring basic molecules. In addition to the existence of a basic molecule in each lattice, each grid may also have readin and readout enzymes. The role of the readin enzyme is that when it receives an external signal, it is activated and will almost simultaneously activate the same basic molecule that coexists with it. Each activated basic molecule will continue to activate its adjacent basic molecules of the same type. (Note: different types of basic molecules will have different degrees of mutual influence, which will be described in detail later). This interaction process continues to the next adjacent basic molecule to form a signal flow on the molecular structure. The above process briefly illustrates how a readin enzyme converts a signal from the outside into a signal for an information-processing neuron. An activated molecule will then enter a reversal phase. During this very short inversion period, the activated molecule cannot be activated again until its inversion period has ended, which will ensure that a single direction of signal flow is maintained. When a stream of basic molecular signal flows is sent to a place with a readout enzyme, the information-processing neuron fires.

During evolutionary learning, the patterns of basic molecules, the readin enzymes, and the readout enzymes, are altered so that an IP neuron has the chance of being trained to be a special input/output converter. This study assumes that three different types of basic molecules have different signal transfer speeds: fast, intermediate, and slow. In addition to the different signal transfer speeds, we also assume that they have different degrees of influence on each other. In general, one basic molecule with a fast transfer speed has less effect on other types of basic molecules. Conversely, a basic molecule with a slow signal transfer speed has a greater effect on other basic molecules. This study utilizes the above two assumptions to construct an information processing architecture with integrated spatiotemporal information. In order to avoid excessive interaction of different types of molecules, this study adds a mechanism of linking proteins (called MAP). Its role is to decide whether to allow interaction between two different types of molecules. During the evolutionary learning process, the distribution pattern of MAPs will also change accordingly to adjust the signal flow pattern within an information processing neuron. 

For example, in Figure 2, the activation of the readin enzyme at location (1,4) will activate the molecule at the same site. The activation of the latter will in turn activate its neighboring molecule of the same type at location (2,4), which in turn activates the molecule at location (3,4). The repetition of the above process will thus trigger a cytoskeletal signal flow along the C_3_ molecules of the fourth column, starting from location (1,4) and running to location (8,4). Likewise, the activation of the readin enzyme at location (3,4) will activate the molecules at the same site. The activation of the latter will in turn activate its neighboring molecule of the same type at location (3,4), which in turn activates the molecule at location (4,4). The repetition of the above process will thus trigger a cytoskeletal signal flow along the C_3_ molecules of the third column, starting from location (1,4) and running to location (8,4). As noted above, a neuron will fire if a readout enzyme is activated.

In Figure 2, there are three signal flows that may possibly combine to activate the readout enzyme. The first is a signal flow on the second column. It may be activated either by the readin enzyme at location (2,2) or by the readin enzyme at location (3,2). The second is a signal flow on the third column, which may be activated by the readin enzyme at location (4,3). The third is a signal flow on the fourth column, which may be activated either by the readin enzyme at location (2,4) or by the enzyme at location (4,4). Any two of the above three signal flows might activate the readout enzyme at location (8,3), which in turn will cause the neuron to fire. However, it should be noted that the neuron might fire at different times, as it might respond to different combinations of signal flows traveling along these fibers. 

The readout enzyme at the (8,3) position can be activated by signal flow from the second, third, and fourth columns. Under current assumptions, it is possible that any two of the three signal streams described above would cause the readout enzyme at (8,3) to be activated. However, it must be emphasized that the combination times of different combined signals are not necessarily the same, resulting in different firing times of a neuron. Indirectly, it changes the start time and rotation angle of the motor it controls (details are explained in the next subsection). A detailed description of how an information processing neuron works can be found in [17,18,19,20].

### 2.3. Application Domain

**Figure 3:**
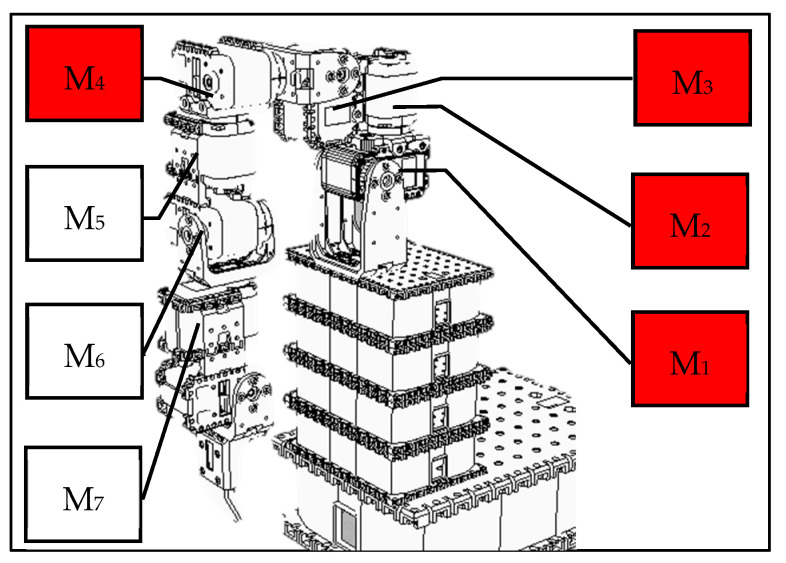
A humanoid robotic arm using V-REP simulation environment.

As mentioned above, the purpose of this study is not to build a state-of-the-art rehabilitation system but to use it as an experimental testbed to build a system that uses intelligent system design to assist in constructing a personalized rehabilitation system. This study uses the V-REP simulation environment to design a humanoid manipulator simulation system (Figure 3). In the design of arm movements, this study only considers some human arm movements that can be roughly imitated by machines. In other words, some human arm movements that are limited by the motor design and assembly that cannot be simulated will not be considered in this study.

After many tests in this study, the following actions can be roughly imitated. The rotation of the single motor 1 can simulate the shrug of the human body; the rotation of the single motor 2, the swing of the arm; the rotation of the single motor 3, the side lift of the arm; and the rotation of the single motor 4, the raise of the arm. For each feasible action, we take the end position (X, Y, Z coordinate values) of the robot arm every 50 milliseconds during the execution of the action. Finally, we link each position throughout the action to create a target trajectory. 

The fitness of each learning cycle is obtained by comparing the motion trajectories produced by the ANM-controlled robotic arm with the target trajectories. The algorithm used in this study is 3D Dynamic Time Warping (DTW for short). The method uses dynamic programming to try to find the maximum similarity between two trajectories (that is, the 3D coordinate values of each point in the movement trajectory of the end point of the robot arm and the movement trajectory it is assigned to learn). In terms of the value of DTW, the smaller its value is, the greater the similarity (i.e., the higher the similarity between two trajectories). The trick to using the DTW algorithm is that a single point on one trajectory can match multiple points on another trajectory. No matter how it is matched, two principles must be followed. The point-to-point matching of different trajectories must keep the original point order on the trajectories. The second is that every point of one trajectory must match at least one point of the other trajectory. For example, for two trajectories of length m and k, an m × k grid can be created, where each grid point (i, j) represents from point i on trajectory one to point j on trajectory two the distance. The detailed algorithm starts at grid point (1, 1). The next step is to increment the value of i or j (or both) by 1 and continue this step until the computation reaches the point (m, k). Since the testbed used in this study is a computer simulation environment, the size of the computer grid is referred to as a unit. When the distance in the computer simulation is constructed, the warp function takes the cumulative distance matrix as input and returns a list of index pairs representing the best warp path. This path represents where the two trajectories have the greatest similarity, and the cumulative distance value is the so-called DTW value. Through the evolutionary learning algorithm, the starting time of different motors, the length of rotation time, and the sequence of motor operation are modified to complete the specified task assignment. 

**Figure 4:**
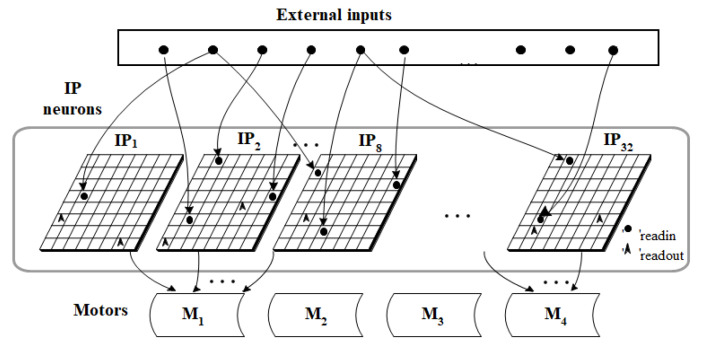
Interface between the ANM system and motors of the humanoid robotic arm.

Figure 4 shows the interface between the IP neurons of the ANM system and the robotic arm motors. In this study, the IP neurons of each sub-network of the ANM system were divided into four groups. Each group controls one motor. For each sub-network, the first neuron in the same group to generate firing behavior was used as the on-time of the motor. A second neuron in the same group that produces firing behavior serves as the motor’s off time. In other words, the time difference between the first two firing neurons represents the length of time the motor is spinning, that is, the angle of rotation of the motor. That is, the time difference between two firing neurons will be converted into the rotation angle of the motor. This study further assumes that the time difference and the motor rotation angle are a sigmoid-like function. After many attempts, the formula currently used in this study is shown in Equation (1).

(1) $$a=\left(\left(\frac{1}{1+e^{-1*\left(15*\Delta t\right)}}\right)-0.5\right)*2-\mathrm{default}\;\mathrm{angle}$$

**Figure 5:**
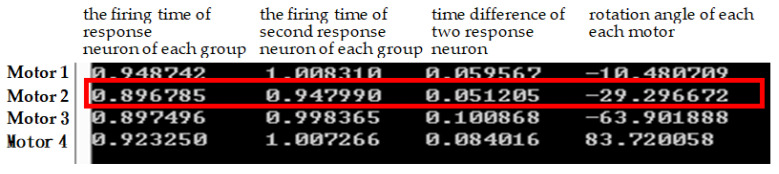
An example of the first response neurons of each group that determine the on/off time of each motor.

Figure 5 shows an example of the first two firing neurons of each group. If motor 2 is used as an example, the neuron’s first trigger time is 0.896785 milliseconds and the second trigger time is 0.947990 milliseconds. The time interval between the triggering of the two neurons is 0.051205 milliseconds. Taking motor 2 as an example, the first firing time of the neuron is 0.896785 milliseconds and the second firing time is 0.947990 milliseconds. The time interval between two neurons firing is 0.051205 milliseconds. Suppose the preset angle value of motor 2 is −80 degrees. If the above values are substituted into Equation (1), the value of Equation (2) is obtained. The whole calculation results in −29.296672. The above results briefly illustrate how to convert the time interval between two neuron firings into motion angles. Basically, the starting time (starting time of action) of each motor is determined according to the value generated by the system. Sometimes the starting of these motors is somewhat sequential, that is, one motor completes its action and then starts the other. However, sometimes it is an overlapping operation, that is, there are two or more motors operating at the same time. For example, from Figure 5, we can see that the start-up time of motor 1 is 0.948742, which is larger than the end time of motor 2’s rotation (0.947990). This means that the start-up time of motor 1 is after motor 2 has completed the turning action. In contrast, the start-up times of motor 3 and motor 4 are 0.9897496 and 0.923250, respectively, and these values are smaller than the end time (0.947990) of the rotation of motor 2. This means that the rotation of motor 3 and motor 4 overlaps with the rotation of the rear stage of motor 2. This means that the three motors (motors 2, 3, 4) are operating simultaneously for certain time periods.

(2) $$a=\left(\left(\frac{1}{1+e^{-1*\left(15*0.051205\right)}}\right)-0.5\right)*2-80=-29.296672$$

## 3. Results

This research uses the ANM system with autonomous learning function as the learning mechanism to establish a robotic arm simulation system that assists people’s arm movement. Through this system, under the guidance of a physician or specialist, the hope of this study is to guide patients through different stages of rehabilitation using an asymptotic approach. However, as to how to generate an asymptotic approach, the recommendation of this study is to have the patient move on a trajectory specified by the physician or specialist during the various stages of rehabilitation. Immediately after the above-mentioned problem is how to control the robot arm in order to generate a specified motion trajectory. The whole system was simulated on the V-REP (Virtual Robot Experiment Platform). How to control the start-up time, rotation angle and motor running sequence of different motors to accomplish the specified task is not a simple problem. 

The following experiments, ranging from simple to complex, were conducted in three stages. The first stage is where the ANM system learns the action of spinning only one motor. The second stage is for the ANM system to learn to solve actions that require the arm to rotate two or more motors (i.e., increase the difficulty of the problem). The final stage is to let the ANM system find the solution on its own with partial rewards. 

### 3.1. Single Motor Action

**Figure 6:**
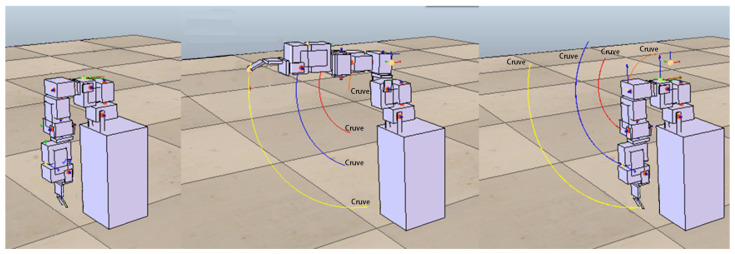
The motion trajectory of the side-lifting action. Each colored line represents the movement trajectory of the motor bearing. In this study, only the fingertip trajectory (in yellow color) of the robotic arm was used.

The goal of this experiment is to expect that the ANM system can learn to control the robotic arm to perform four simple actions: shrugging, swinging, side-lifting, and up-lifting. When motor 1 rotates 25 degrees alone (the remaining three motors remain stationary), the robotic arm will perform a similar shrugging motion. When motor 2 alone rotates 40 degrees (the other three motors remain stationary), the robotic arm will make a movement similar to a swinging arm. By analogy, when motor 3 rotates 100 degrees and motor 4 rotates 150 degrees, the robotic arm will perform side-lifting and up-lifting actions respectively. Figure 6 shows the motion trajectory of the side-lifting action. For each action, this study first recorded the trajectory of each action as the target trajectory and then asked the robotic arm (controlled by the ANM system) to perform similar actions to the specified target trajectory. 

**Figure 7:**
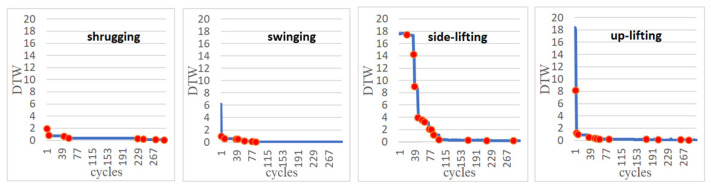
Learning progress of the ANM system such that the four motors started at the same time (Red dots represent where learning performance has improved).

For each of the experiments, we stopped the ANM system learning when the learning progress was almost obviously slow (approximately after about 300 cycles). Figure 7 shows that in each action, the DTW decreases as the number of learning increases. Obviously, it can be seen that the DTW decreases rapidly in the early stage of learning, while it appears to be significantly slower in the later stage of learning. However, in the learning process, it still shows the phenomenon of continuous learning (the red dot in each figure represents when the DTW changes). 

From Table 1, we can see that the rotation angle of each motor learned by the system is very close to the preset angle. After 300 times of learning, the DTW is reduced to less than 0.15. This result shows that under the control of the ANM system, the robot arm can learn the specified movement trajectory. In other words, the ANM system can use a self-learning method (that is, to find a set of angles at which each motor operates) to generate a robot arm movement close to the specified movement trajectory. It is added here that in terms of machine learning, the number of learning times of 300 only represents the time required for computer simulation. In terms of the continuous learning ability presented by the ANM system, when the allowed simulation time is longer, the moving path it can find will be closer to the specified path. Basically, this is the processing work on the computer side, and it has little to do with the actual work in the clinic.

### 3.2. Multiple Motor Actions

**Figure 8:**
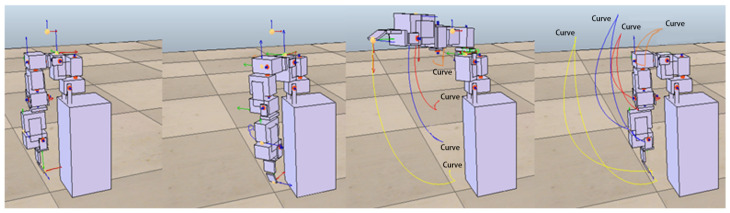
The motion trajectory of the swinging→side-lifting action. The robotic arm first performs the swinging motion, then performs side-lifting motion, and finally returns to the original starting state.

**Figure 9:**
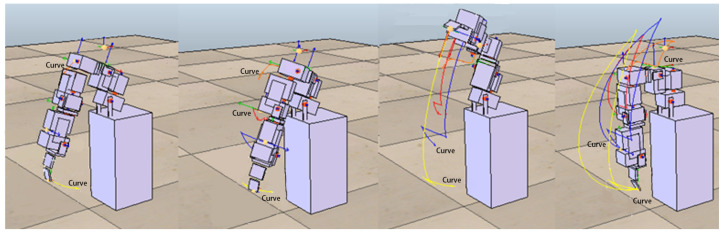
The motion trajectory of the shrugging→swinging→side-lifting action. The robot arm performs three actions in sequence, including shrugging, swinging, and side-lifting.

In the following experiments, we further increase the complexity of actions that require two or three motors to complete. The approach adopted in this study is to set up more complex actions from different combinations of the above-mentioned four simple actions. Combinations of actions using two motors included shrugging to swinging (to be denoted as shrugging→swinging), shrugging to side-lifting, shrugging to up-lifting, swinging to side-lifting, side-lifting to up-lifting, up-lifting to swinging. Figure 8 shows the motion trajectory of the robotic arm first performing the swinging motion, then performing side-lifting motion, and finally returning to the original starting state. Combinations of actions using three motors included shrugging to swinging to side-lifting (to be denoted as shrugging→swinging→side-lifting), shrugging to swinging to up-lifting, shrugging to side-lifting to up-lifting, swinging to side-lifting to up-lifting. Figure 9 shows the motion trajectory of the robotic arm performing three actions in sequence, including shrugging, swinging, and side-lifting. All motors in the above actions must follow a certain sequence to reach the target trajectory. For each of the above complex actions, this study first records the trajectory of each combined action as the target trajectory and then asks the ANM system to control the robotic arm to carry out similar actions. 

**Figure 10:**
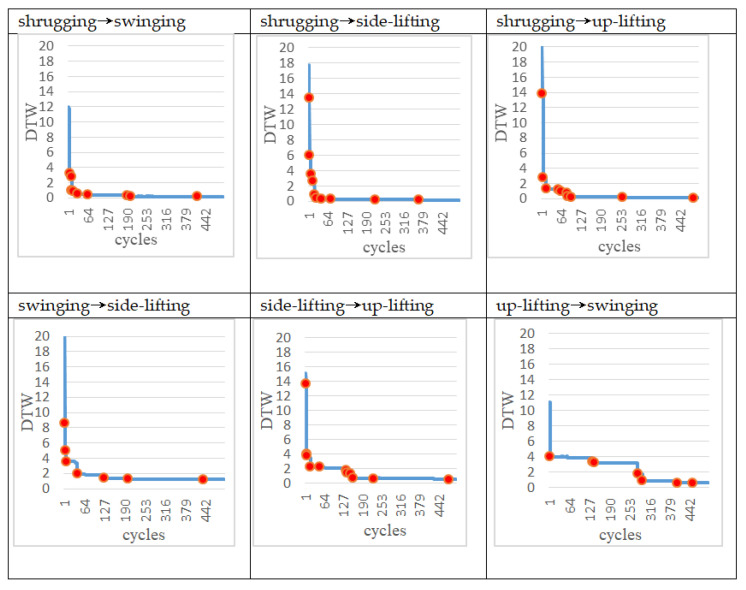
Learning progress of the ANM system for complex actions using two motors.

**Figure 11:**
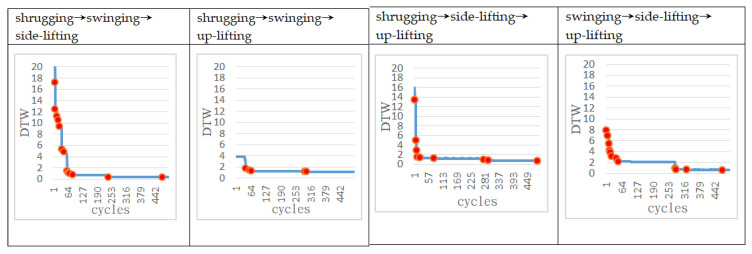
Learning progress of the ANM system for complex actions using three motors.

Figure 10 and Figure 11 show that the DTW decreases with the increase of the number of learning cycles for the complex actions using two and three motors, respectively. From Table 2, we can see that under the control of the ANM system, no matter which action it is, its final DTW value is significantly improved (all less than 1.25) when compared with the initial value. This means that the robotic arm can automatically learn to find out the start-up time and operating angle of each motor to find the motion close to the target trajectory. More specifically, if we compare the learned results of all motions of the four motors (the predetermined learned angle of each motor and the learned rotation angle), motor 2 has the largest difference value. In other words, it is relatively difficult for motor 2 to learn the predetermined rotation angle. This phenomenon is particularly evident in two experiments (the arm swinging movement and the shrugging→swinging swing movement). That is, although the ANM system can learn to approach the target trajectory, the angle used by each motor is not the same as the preset angle (especially motor 2). The inference of this result is that the change of the motor angle of motor 2 has less effect on the change of the entire manipulator operating range, that is, the difference of its rotation angle does not significantly affect the learning result. Because of this, the role it plays is also relatively insignificant. In the process of learning, the requirement of whether it has learned the predetermined angle in advance is relatively low, resulting in a relatively high angle difference. However, it is interesting that the ANM system designed in this study can find another feasible solution through autonomous learning. 

### 3.3. Partially Rewarded Actions

In the following experiments, instead of giving the entire trajectory, several target points are given to test whether the trajectory generated by the robot arm (controlled by the ANM system) is close to these target points. In other words, we hope that the ANM system can automatically adjust the starting time, sequence, and angle of the four motors to find the closest path to all the target points. From a certain point of view, due to the reduction of the number of learning reference target points, the difficulty for the system to solve these problems can be relatively high. In addition, since the learning target of this experiment is no longer a target trajectory, the DTW evaluation method used in the previous experiments cannot be applied. The evaluation method of this experiment is based on the sum of the Euclidean distances between the points closest to the entire trajectory and each target point. There are two methods to select the target points. The first is to randomly take 3, 5, and 7 points, respectively, from the target movement trajectory in the previous experiment as target points. For each of the three target point numbers (3, 5, 7), two experiments were performed. The choice of target point for each experiment was randomly selected from a single motor action or two motor actions. The second method is to arbitrarily select points 3, 5, and 7 as target points within the possible operating range of the trajectory in the previous experiment. In order to understand the problem-solving ability of the ANM system, this experiment increases the number of experiments from two to three for each of these three target point numbers. Similarly, the choice of target point for each experiment was randomly selected from a single motor action or two motor actions.

**Figure 12:**
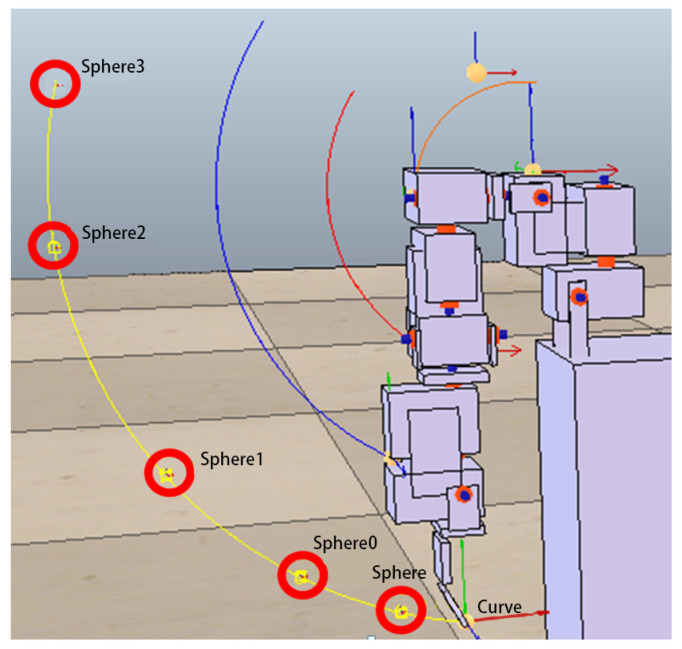
An example of the learning result of the ANM system when five target points are randomly selected from the trajectory of the side-lifting action.

**Figure 13:**
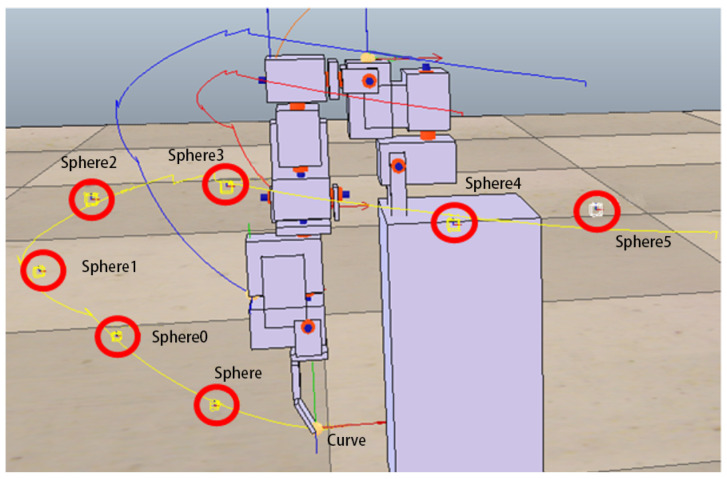
An example of the learning result of the ANM system when seven target points are arbitrarily selected from the trajectory of the side-lifting→swinging action.

**Figure 14:**
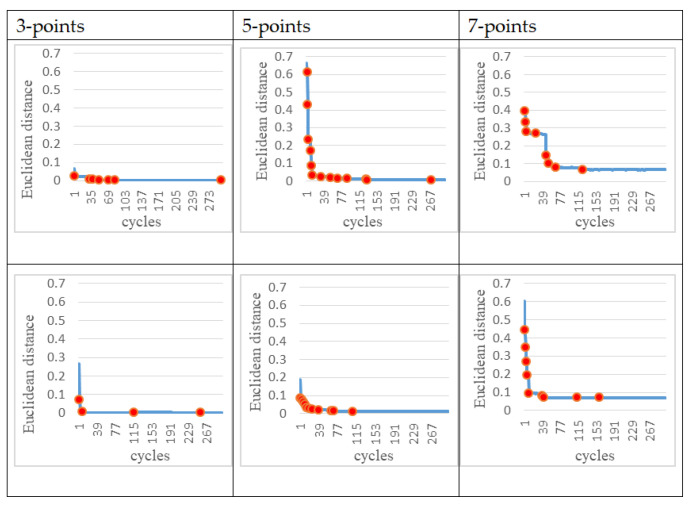
Learning progress of the ANM system for the target points taken from the target trajectories.

**Figure 15:**
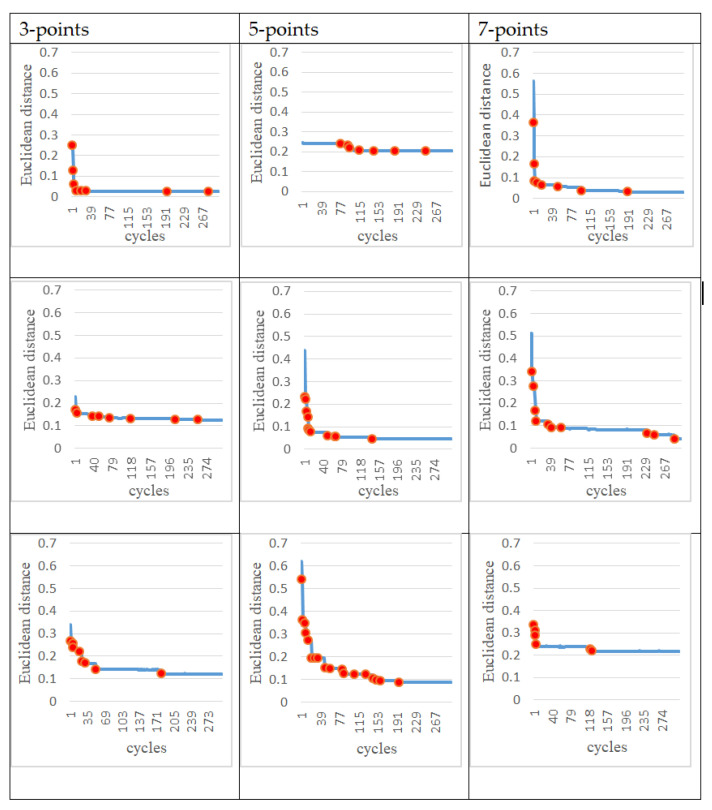
Learning progress of the ANM system for the target points taken from the possible range of the target trajectories.

The experimental results at this stage show that the ANM system can find trajectories close to each target point without knowing in advance how it works. In other words, although the movement of the robotic arm cannot accurately pass through every target point, it can find a trajectory close to each target point. Figure 12 shows an example of the optimal movement trajectory obtained through the ANM system learning when five target points are randomly selected using the first target point selection method on the trajectory of the side-lifting action. Figure 13 shows an example of the optimal motion trajectory learned by the ANM system when seven target points are arbitrarily selected using the second target point selection method on the trajectory of the side-lifting→swinging action. Figure 14 showed the learning progress of the ANM system for the target points taken from the target trajectories whereas Figure 15 for the target points taken from the possible range of the target trajectories. It is added here that the results presented in Figure 12, Figure 13, Figure 14 and Figure 15 are only experimental results obtained so far. The experiments at this stage of this study are a preliminary exploration of using partial information to establish the functionality of the robotic arm movement trajectory. Therefore, only partial experiments have been carried out at present (i.e., not all possible experiments have been completed in a systematic experimental manner). 

The above results have considerable significance in assisting those in need of rehabilitation. We all know that what appears to be a fairly simple movement to a healthy person can be quite difficult for a patient. During the recovery process, the patient may have to undertake some kind of incremental rehabilitation improvement (progressive rehabilitation). This study is making efforts in this direction by using the method of providing partial target points to let the system find a feasible trajectory. The experimental results in this part prove that the autonomous learning system can find the trajectories approaching these target points in the self-learning way. In the future, through the physician’s assessment and advice, he or she can set up these target points for the specific needs of the patient, and the system can provide suggested arm movement trajectories under these conditions.

## 4. Discussion

This study uses the V-REP simulation system to construct a simple human-like motion manipulator operating platform and learns the different motions of the manipulator through the ANM system. The suitability (fitness) of each learning cycle is to compare the similarity between the motion trajectories generated by the molecular-nervous-system-controlled robot and the target motion trajectories. 

The results show that the ANM system can adjust the start-up time, the rotation time, and the running sequence of different motors by means of self-learning until a path similar to the target trajectory is imitated. Most of the learning results show that the start-up time of different motors, the length of the rotation time, and the sequence of motor operation are very close to the previously set values. However, in some cases, the values obtained by the ANM system differ considerably from those previously set.

The ANM system can search for a suitable combination of motor starting angle and time to learn to move towards a similar target trajectory. The most commendable thing is that the ANM system can find not only the coordinated method of a group of motors to achieve the target trajectory but also use different search methods. However, in some cases, the values (angles of rotation of certain motors) obtained by the ANM system are quite different from the previously set values. The experimental results show that the system can search for a suitable combination of rotational angles and motor starting times by itself, and learn to advance toward a trajectory similar to the target. The most commendable thing is that the ANM system can find not only the coordinated method of a group of motors to achieve the target trajectory but also use different search methods. The ANM system has these capabilities mainly because it has, as mentioned earlier, a close structure/function relationship. Through this relationship, the ANM system can accumulate the results learned before. Without falling into the best solution of the area, it can search for other possible directions to solve the problem.

## 5. Conclusions

The richness of the ANM system described in this paper allows us to use evolutionary learning methods to perform various experiments on complex problems. The richness of the ANM system described in this paper enables us to perform various experiments on complex problems using evolutionary learning methods. The current way of operating the system is that at a certain time, we only allow evolutionary learning at a certain level, while other levels of learning are turned off. When learning for a fixed period of time, the system will turn off this level of learning and turn on other levels of learning. In this way, the system opens up each level of learning in turn. This system structure allows for two different levels of information processing: the macro-level and the micro-level. Macro-level information processing refers to combining (or manipulating) information processing neurons with different signal integration capabilities in an evolutionary learning manner to complete a harmonious function. Micro-level information processing refers to changing the signal integration capabilities of information processing neurons in an evolutionary learning manner that occurs at the level that initiates cytoskeletal signaling flow, the level of cytoskeletal signaling pathways, and the level of cytoskeletal signaling integration. This organization enables the interaction of evolutionary processes at different organizational levels to generate solutions to complex problems. Most importantly, the openness of evolutionary processes can always be increased by placing more structural features and local interaction rules in mutation-selection operations. Our experiments with the ANM system show that it provides significant computational synergy for the integration of different information processing modalities. The internal dynamics of the information-processing neurons we choose play a very important role. The ANM system uses weak interactions, redundant properties, compartmentalization, and other properties to build signal integration patterns to establish the gradual transformation of “structure/function” relationships. Using this relationship, it allows the entire system to use mutation and selection to move forward in the direction of completing the task. 

As mentioned above, this study achieved preliminary good results in using the ANM system to control the robotic arm to generate target trajectories in a self-learning manner. In the future, in order to prove the feasibility of its application, this study must systematically demonstrate its actual function through a series of experiments. For example, in terms of specifying motion trajectories and target points, this study can gradually increase the number of target points and the complexity of motion trajectories. In addition, this study may consider further cooperation with the hospital on rehabilitation exercise such as establishing a systematic trajectory database for the different rehabilitation needs of different patients. It could even be used to control exoskeleton devices in the future when the entire system is fully functionally tested. If this can be done, it could not only be used for rehabilitation but also increase the possibility of developing smart prosthetics.

## Figures and Tables

**Table 1:** The experimental results of motors with the same starting times.

	Angel of Motor 1		Angel of Motor 2		Angel of Motor 3		Angel of Motor 4		DTW (Three-Axis Euclidean Distance)
Action	Assigned	Learned	Assigned	Learned	Assigned	Learned	Assigned	Learned	(from→to)
shrugging	−25	−25	0	0	0	0	0	0.9	1.917→0.057
swinging	0	0	−40	−39.9	0	0	0	0	6.214→0.021
side-lifting	0	0	0	0	−100	−100	0	0.5	17.807→0.061
up-lifting	0	0	0	0	0	0	150	149.5	18.121→0.148

**Table 2:** The experimental results of multi-motor actions.

Complex Actions	Motor 1		Motor 2		Motor 3		Motor 4		DTW
(from→to)	Assigned	Learned	Assigned	Learned	Assigned	Learned	Assigned	Learned	(from→to)
shrugging→swinging	−20	−20.1	−40	−29.1	0	−0.6	0	1.1	11.594→0.188
shrugging→side-lifting	−25	−25	0	0	−90	−89.6	0	0.2	17.731→0.177
shrugging→up-lifting	−25	−24.5	0	−0.1	0	−0.6	150	150	20.296→0.145
swinging→side-lifting	0	−13	−40	−32.5	−100	−89.5	0	1.2	26.013→1.234
side-lifting→up-lifting	0	0	0	−5.6	−100	−99.5	50	45	15.781→0.512
up-lifting→swinging	0	−0.3	−40	−40.3	0	0	100	101.2	11.231→0.655
shrugging→swinging→side-lifting	−25	−25	−40	−40.6	−90	−90	0	0.2	22.795→0.345
shrugging→swinging→up-lifting	−20	−15.5	−40	−43.4	0	−6	100	96	3.981→1.099
shrugging→side-lifting→up-lifting	−25	−24.5	0	−0.7	−90	−90.7	150	146.7	15.947→0.727
swinging→side-lifting→up-lifting	0	0	−40	−41	−90	−89.9	100	98.8	7.956→0.586
